# Life Uncertain

**Published:** 1878-08

**Authors:** 


**Life Uncertain.**—Life is beautifully compared to a fountain filled up by a thousand streams that perishes if one be dried. It is a silver cord twisted with a thousand strings that parts asunder if one be broken. Frail and thoughtless mortals are surrounded by innumerable dangers, which make it much more strange that they escape so long than that they almost all perish suddenly at last. We are encompassed with accidents every day to crush the moldering tenement that we inhabit. The seeds of disease are planted in our constitution by the haud of nature. The earth and the atmosphere, whence we draw our life, are impregnated with death—health is made to operate its own destruction. The food that nourishes the body contains the elements of its decay ; the soul that animates it by a vivifying fire, tends to wear it out by its action ; death lurks in ambush along our path. Notwithstanding this is the truth, so palpably confirmed by daily examples before our eyes, how little do we lay it to heart. We see our friends and neighbors perishing around us, but how seldom does it occur to our thoughts that our knell shall, perhaps, give the next fruitless warning to the world?

There is something eminently tragic in the lives of almost all the princes and princesses of the great Muscovite Kingdom. Some die by the dagger, some by poison ; some are dropping off suddenly in a mysterious manner, and others are ailing for years under the influence of a malady of which nobody knows the cause, and for which no physician can give advice. There has scarcely been one sovereign of Russia whose death appeared quite natural. Even the predecessor of the present Czar died with a mysterious suddenness, although he was one of the healthiest and strongest men in Europe, hardened like a mountaineer, simple and frugal in his habits, and accustomed to fatigue and the extremes of heat and cold.

